# 免疫检查点抑制剂在小细胞肺癌治疗中的临床研究进展

**DOI:** 10.3779/j.issn.1009-3419.2019.04.07

**Published:** 2019-04-20

**Affiliations:** 650032 昆明，昆明医科大学第一附属医院检验科，云南省检验医学重点实验室，云南省实验诊断研究所 Department of Clinical Laboratory, the First Afliated Hospital of Kunming Medical University; Yunnan Institute of Experimental Diagnosis; Yunnan Key Laboratory of Laboratory Medicine, Kunming 650032, China

**Keywords:** 免疫检查点抑制剂, 小细胞肺癌, PD-1, PD-L1, CTLA-4, Immune checkpoint inhibitors, Small cell lung cancer, PD-1, PD-L1, CTLA-4

## Abstract

随着人们对肿瘤免疫的深入研究，以免疫检查点抑制剂（immune checkpoint inhibitor, ICI）为重要代表的肿瘤免疫疗法在实体肿瘤方面取得了重大突破。小细胞肺癌（small cell lung cancer, SCLC）占所有肺癌的15%-20%，恶性程度高，早期易转移，暂缺乏有效的治疗策略。免疫检查点抑制剂的出现给SCLC的治疗带来了新的希望，多项临床试验显示了程序性死亡受体/配体-1（programmed death receptor 1/ligand 1, PD-1/L1）和细胞毒性T淋巴细胞抗原-4（cytotoxic T-lymphocyte antigen 4, CTLA-4）对SCLC的持久的疗效和临床活性，但目前其有效性和安全性暂不十分确切，且有效预测免疫治疗疗效的标志物一直没有定论。为此，本文综述了免疫检查点抑制剂及其相关标志物在SCLC治疗中的研究进展，以探讨SCLC免疫治疗的价值以及存在的问题与挑战，为进一步改变SCLC治疗策略的临床实践与研究提供理论依据。

## Abstract (en)

With the in-depth study of tumor immunity, immunotherapy represented by immune checkpoint inhibitors has made a great breakthrough in solid tumors. Small cell lung cancer (SCLC) accounts for about 15%-20% of all lung cancers, with high malignancy, early metastasis and lack of e?ective treatment strategy. Te appearance of immune checkpoint inhibitors brings new hope for SCLC. Several clinical trials have demonstrated the persistent efcacy and clinical activity of the programmed death receptor/ligand 1 (PD-1/L1) and cytotoxic T-lymphocyte antigen 4 (CTLA-4) in the treatment of SCLC. However, its efcacy and safety are not very accurate, and the markers that can e?ectively predict the efcacy of immunotherapy have not been concluded. In this paper, for further changing the treatment strategy of SCLC clinical practice and providing theoretical basis of research, we reviewed the progress of immune checkpoint inhibitors, related markers in the treatment of SCLC by exploring the value, problems and challenges of immunotherapy in SCLC.

## 1 前言

2018年全球癌症统计显示肺癌仍然是最常见的恶性肿瘤（占总癌症患者的11.6%），也是第一大癌症相关死因（占所有癌症死因的18.4%）^[1]^。由于控烟不力、青少年吸烟人数剧增等诸多因素，我国肺癌发病率更是呈现有增无减的趋势。小细胞肺癌（small cell lung cancer, SCLC）是一种高侵袭性的神经内分泌肿瘤，占肺癌总数的15%-20%^[2]^，恶性程度高，早期易发生转移，手术一般只对大约1%的患者有益^[3]^。治疗SCLC的一线疗法是以铂类为基础的系统化学治疗，有良好的初始有效率，然而，绝大多数的患者都会在1年内复发和耐药^[4-6]^。根据美国国家癌症综合网（National Comprehensive Cancer Network, NCCN）指南，美国食品与药品监督管理局（Food and Drug Administration, FDA）批准的唯一的第二线疗法是拓扑替康，但静脉滴注拓扑替康的中位有效期（duration of response, DOR）也只有3.3个月，疗效无法令人满意，且会出现严重的中性粒细胞减少症、贫血、血栓发生率升高等毒副作用^[7-9]^。研究表明，免疫系统与肿瘤之间相互作用导致的肿瘤免疫逃逸是肿瘤发展的一个关键因素。肿瘤通过灭活各种能促进细胞死亡的T细胞毒性途径来避免免疫监视。在T淋巴细胞识别肿瘤抗原的过程中，免疫检查点信号通路具有极其重要的作用。基于此，近几年展现出良好前景的肿瘤免疫疗法无疑是人类肿瘤治疗史上的一座重大里程碑，目前已经上市的PD-1单抗、PD-L1单抗和CTLA-4单抗为代表的ICI在很多实体肿瘤（特别是霍奇金淋巴瘤和黑色素瘤）治疗中取得重大突破。既往研究^[10, 11]^显示SCLC因其肿瘤突变负荷（tumor mutation burden, TMB）高、含较多效应T细胞，这提示免疫检查点抑制剂可能会对SCLC产生很好的疗效。

《ASCO年度报告：2018临床肿瘤学进展》^[12]^指出，在临床试验中，接受ICI治疗的患者较标准化疗的患者生存期更长。但在临床试验中发现，仍有部分患者在接受免疫治疗时没有反应或疾病仍在进展。且随着免疫抑制剂的广泛批准使用，越来越多的肿瘤患者使用这一类药物，其相关的并发症逐渐浮出水面。因此，如何优先筛选出那些更可能受益于免疫治疗的患者尤为重要。本文将对免疫检查点抑制剂及其相关标志物在SCLC治疗中的研究进行综述。

## 2 免疫检查点抑制剂在SCLC的临床应用

### 2.1 CTLA-4抑制剂

CTLA-4是表达在细胞毒性T淋巴细胞上的一种免疫反应负调节器，通过与抗原提呈细胞上表达的B7-1（CD80）/B7-2（CD86）结合，进而对CTL的激活产生负向调控^[13]^。CTLA-4抑制剂是临床最早开展研究的ICI，其能与CTLA-4蛋白结合，阻断CTLA-4介导的免疫抑制作用，激活CTL参与抗肿瘤免疫应答。首个针对SCLC的免疫靶向治疗药是完全针对CTLA-4蛋白的人源化单克隆抗体——伊匹单抗（Ipilimumab）。Reck教授等^[14]^在CA184-041 Ⅱ期临床试验（NCT00527735）中，采用Ipilimumab联合紫杉醇/卡铂方案作为一线或维持治疗广泛期SCLC（ES-SCLC），将纳入的130例ES-SCLC患者随机分为三组：ipilimumab同步化疗组（先予4个疗程ipilimumab/紫杉醇/卡铂，后改用2个疗程安慰剂/紫杉醇/卡铂）、ipilimumab阶段治疗组（先予2个疗程安慰剂/紫杉醇/卡铂，后改为4个疗程ipilimumab/紫杉醇/卡铂）及对照组（最多6个疗程安慰剂/紫杉醇/卡铂）。结果显示，通过免疫相关反应标准（immune-related response criteria, Irrc）分析，ipilimumab阶段治疗组的客观反应率（objective response rate, ORR）最佳为71%，同步化疗组的ORR为49%，对照组为53%；ipilimumab阶段治疗组免疫相关无进展生存期（immune-related progression-free survival, irPFS）得到改善（HR=0.64; *P*=0.03），而同步化疗组（HR=0.75; *P*=0.11）没有显著改善；三组的中位PFS（5.2个月*vs* 3.9个月*vs* 5.2个月）、总生存期（overall survival, OS）中位数（12.9个月*vs* 9.1个月*vs* 9.9个月）均无统计学差异；Ⅲ级-Ⅳ级免疫相关不良反应（immune-related adverse events, irAEs）在ipilimumab治疗组中更常见（50% *vs* 43% *vs* 30%）。进一步的ipilimumab联合化疗的验证性评估研究（CA184-156 Ⅲ期试验）采用先予3个疗程卡铂/依托泊苷化疗，然后每12周予ipilimumab维持治疗。结果显示，与单纯化疗组相比，OS（11个月*vs* 10.9个月，HR=0.94）、中位PFS（4.6个月*vs* 4.4个月，HR=0.85）没有明显改善^[15]^。Ⅲ期研究未能进一步验证Ⅱ期试验的结果，Pakkala^[16]^分析原因可能为：在Ⅱ期试验（卡铂/紫杉醇）和Ⅲ期试验（卡铂/依托泊苷）中使用的化疗药物不同；纳入的病例之间存在差异；Ⅱ期研究中的有效率是基于irPFS的，而这个新型的观察终点目前暂未被验证能替代OS；最重要的是，由于CTLA4信号与免疫应答的相对非特异性激活阶段更相关，ipilimumab对肿瘤特异性免疫调节作用可能是有限的。Murray教授^[17]^考虑可能是相关肿瘤抗原表达不足、给药顺序及联合化疗药物选择的不同、ipilimumab联合化疗并没有引起肿瘤微环境中的T细胞的活化，以及化疗限制了T细胞的活化与增殖，没有激起有效的抗肿瘤免疫。

### 2.2 PD-1/PD-L1抑制剂

PD-1为表面跨膜糖蛋白受体，属于B7-CD28受体家族成员之一^[18]^。PD-1及其2个配体[PD-L1（B7-H1）和PD-L2（B7-DC）]对T细胞的激活具有负向调控作用。当PD-1与PD-L1相结合后，可通过下调磷脂酰肌醇3-激酶信号通路，减少*γ*-干扰素（interferon-*γ*, IFN-*γ*）等细胞因子分泌，抑制T细胞激活。PD-1/PD-L1单抗通过阻断PD-1与其配体的相互作用进而激活T细胞，产生抗肿瘤免疫。

纳武单抗（nivolumab）是最早被FDA批准上市的针对PD-1的人源化单克隆抗体，同时是我国首个获批上市的免疫肿瘤治疗药物。基于两个标志性的临床试验（Checkmate017、Checkmate057），2015年nivolumab相继被美国FDA批准用于难治或复发的晚期鳞状和非鳞状非小细胞肺癌的二线治疗^[19, 20]^。Ready教授在Checkmate032研究^[21]^中报道了Nivolumab单药治疗复发SCLC，中位随访期为28.3个月，ORR为11.9%，DOR为17.9个月；随访第6个月时，17.2%患者达到无疾病进展生存；12个月和18个月OS分别为28.3%和20.0%，Ⅲ级-Ⅳ级治疗相关不良事件发生率为11.9%。这说明nivolumab单药治疗作为复发SCLC的第三线或更晚的治疗方法，具有持久的疗效和很好的耐受性。一项关于nivolumab单药与化疗比较，治疗复发SCLC的Checkmate331 Ⅲ期研究（NCT02481830）正在进行中，研究结果可能为SCLC患者提供更多新的治疗选择。

派姆布罗珠单抗（pembrolizumab）是一种高选择性的、人源化IgG4型单克隆抗体。2017年美国FDA批准pembrolizumab联合培美曲赛（alimta）与顺铂或卡铂一线治疗转移性非鳞状非小细胞肺癌患者^[22]^。Ott等^[23]^在Checkmate028 Ib期研究（NCT02054806）中纳入PD-L1阳性、标准化疗无法缓解的ES-SCLC患者，给予pembrolizumab单药治疗（10 mg/kg, *iv*, *q2w*），对获得疾病控制的患者继续治疗24个月或直至疾病进展或无法耐受毒副作用。主要终点为安全性、耐受性和客观反应率。该研究更新的最新结果显示：中位随访时间为9.8个月；24例患者中1例达到完全缓解，7例达到部分缓解，ORR为33%；常见的不良反应为无力（7例）、咳嗽（6例）、疲乏（7例）；2例患者出现Ⅲ级-Ⅴ级治疗相关副作用。该研究提示pembrolizumab治疗数据和既往其他癌种类似，缓解呈现长久持续，但仍不可避免地出现免疫相关毒副作用。此外，多项正在进行的研究如pembrolizumab联合同步放化疗治疗初治SCLC的Ⅰ期临床试验（NCT02402920）、pembrolizumab联合紫杉醇治疗难治性SCLC的Ⅱ期临床试验（NC702551432）、pembrolizumab联合P13K抑制剂治疗复发耐药SCLC的研究（NCT02646748）等将会提供更多pembrolizumab治疗SCLC的结果。

作为免疫检查点抑制剂的重要一员，Atezolizumab（阿特朱单抗）是最早开始SCLC领域研究的PD-L1抑制剂。Sequist等^[24]^开展的一项Atezolizumab治疗包含SCLC在内的多种实体瘤的Ia期研究，研究纳入17例SCLC患者，65%的患者既往接受3线以上治疗，以免疫相关反应评价标准ORR为24%，中位PFS：1.5个月（95%CI: 1.2-2.7），中位OS：5.9个月（95%CI: 4.3-20.1），毒副作用方面：11例患者发生Ⅰ级-Ⅱ级治疗相关性不良反应，2例发生Ⅲ级以上治疗相关不良反应，1例患者因Ⅲ级肺炎停药，1例患者发生Ⅴ级肝功能衰竭。初步的结果显示Atezolizumab治疗ES-SCLC具有良好的耐受性。目前正在进行的NCT03041311试验旨在探讨应用卡铂、依托泊苷和阿特朱单抗（E/P/A）一线治疗初诊的ES-SCLC的疗效；另一项卡铂联合依托泊苷联合或不联合PD-L1抑制剂（IMpower133）治疗初诊的ES-SCLC的研究（NCT02763579）预计2019年8月完成；NCT03540420、NCT03262454等试验也正处于招募阶段，我们期待更多令人鼓舞的研究结果，为SCLC患者带来新的曙光。

### 2.3 PD-1/PD-L1抑制剂联合CTLA-4抑制剂

PD-l/PD-Ll单抗和CTLA-4单抗可以通过不同途径的联合抑制将更有效的发挥抗肿瘤免疫作用。Antonia等^[25]^开展的Checkmate032 Ⅰ期/Ⅱ期试验旨在评估nivolumab±ipilimumab（N±I）用于复发性SCLC的疗效和安全性。该研究纳入213例晚期SCLC一线或多线含铂双药化疗失败后的患者，其中98例患者接受N单药（3 mg/kg, *q2w*），115例患者接受N+I联合方案（61例患者接受N 1 mg/kg+I 3 mg/kg；54例患者接受N 3 mg/kg+I 1 mg/kg，*q3w*，4周期后，N 3 mg/kg, *q2w*维持治疗），主要研究终点是ORR。结果显示N3单药组ORR为10%、联合组Nl+I3为23%、N3+Il为19%；Ⅲ度-Ⅳ度治疗相关不良事件在N3单药组发生率为13%，N1+I3组为30%，N3+Il组为19%。N3+Il组获得7.9个月的OS，2年生存率达30%。无论N单药还是N+I联合，都显示出了较好的抗肿瘤活性、持久的反应和可控的安全性。这些发现提示，针对PD-1/PD-L1和CTLA-4免疫检查点通路的治疗在改善患者预后方面可能是有效的。

基于Checkmate-032试验的良好结果，NCCN建议将nivolumab±ipilimumab作为SCLC的二线治疗选择方案^[26]^。Checkmate032试验的初步结果带来了新曙光，更多的临床试验将回答关于什么时候联合使用免疫疗法最佳，应该在其他治疗之前还是后的问题。目前正在进行的多项试验，包括nivolumab或nivolumab与ipilimumab联合应用于以铂类为基础的一线化疗后的广泛期SCLC（ED-SCLC）的随机、多中心、双盲Ⅲ期研究（Checkmate-451, NCT 02538666）和由十大癌症研究联盟（BTCRC）开展的nivolumab、ipilimumab和普那布林（plinbulin）应用于复发SCLC患者的Ⅰ期/Ⅱ期研究（NCT03575793），预计2021年完成。

## 3 SCLC免疫检查点抑制剂治疗的疗效预测标志物

大量临床试验结果显示免疫检查点抑制剂在SCLC治疗中表现出较为满意的持久性和有效性。尽管看到了一些持久的好处，但大多数未选定的SCLC患者对检查点抑制剂没有反应。且随着上述药物的广泛批准使用，越来越多的肿瘤患者使用这一类药物，免疫性心肌炎、肝损害等相关的并发症及毒副作用逐渐浮出水面^[27, 28]^。因此建立有效的生物标志物来预测对治疗的反应是追求个性化免疫治疗的一个关键目标，可最大限度地提高治疗效果和减少不良事件的严重程度。

### 3.1 PD-L1作为疗效预测标志物的若干问题

PD-L1作为SCLC生物标志物的作用一直存在争议。初步研究发现，SCLC中PD-L1的表达较低。然而，最近的Keynote-158 Ⅱ期研究^[29]^表明，PD-L1在大约40%的SCLC中可以检测到，并且可以预测pembrolizumab的临床活性。在Checkmate-032 Ⅰ期/Ⅱ期临床试验中nivolumab单药治疗组结果没有显示PD-L1表达的临床活性有显著差异，PD-L1阴性患者的反应较高，nivolumab+ipilimumab联合试验中观察到PD-L1阳性患者却没有反应。此外，免疫组织化学染色检测PD-L1在SCLC中的表达存在许多技术和生物学问题：定义PD-L1在肿瘤中的阳性率受诸如测量PD-L1表达的细胞类型、肿瘤表达的定位与分布、用于评价PD-L1阳性的截止阈值的影响^[30]^。缺乏一种标准的方法来测量PD-L1的表达或对PD-L1的整体表达如何定义有明确的共识，这些将导致无法解释结果和确定最佳阈值，限制了PD-L1作为患者选择的预测生物标志物的有效性^[31-33]^。

### 3.2 TMB或许能更好预测临床活性和预后

体细胞突变是获得性突变，是生长发育过程中或受外界因素影响的后天突变。TMB是肿瘤基因组去除胚系突变后的体细胞突变数量，具体是指肿瘤组织内所评估基因的外显子编码区每兆碱基中发生置换、插入和缺失突变的总数，一般以非同义突变总数量表示^[34]^。研究^[35]^表明，通过检测TMB肿瘤基因组编码区的突变总数可得出，TMB越高，T淋巴细胞可识别的新抗原越多，免疫杀伤活性越强，提示免疫治疗效果可能会更好。Hellmann等^[36]^在2018年的一项关键研究中对401例接受nivolumab±ipilimumab的治疗ES-SCLC患者中的211例（53%）进行了详细的生物标志物分析，通过全外显子测序，69例（27%）为高TMB（每个肿瘤≥248个突变），而73%为低/中度TMB。令人惊讶的是，在TMB高的患者中，nivolumab治疗组的1年OS为35%，nivolumab+ipilimumab治疗组，1年OS为62%，ORR达到21.3%，N-/+I治疗的低/中度TMB患者，OS仅为20%-26%，ORR为4.8%。这提示TMB可作为免疫检查点抑制剂治疗SCLC疗效的预测标志物。然而，2017年ASCO一项研究^[37]^分析了实体瘤的突变负荷，14种实体瘤中具有高TMB的SCLC（99例）的比例仅为5%；关于SCLC和大细胞神经内分泌肺癌的TMB和基因改变的研究中发现，SCLC TMB的中位数为9 mut/Mb，第90百分位数的TMB为19.6 mut/Mb^[38]^。这在某种程度上可能会限制了TMB预测SCLC免疫治疗疗效的价值，但目前研究数据较少，TMB能否成为筛选适合的SCLC接受免疫抑制剂治疗的标志物需要更深入的研究。

TMB作为预测生物标记物在SCLC患者的其他正在进行的和其他计划中的研究有：nivolumab±ipilimumab与Rova-T（DLL4定向抗体药物结合物）的1期/2期试验（NCT 03026166）、一项大型的评估nivolumab±ipilimumab，以及各种免疫治疗药物相互结合和化疗治疗SCLC的CheckMate 451研究（NCT 02538666）。

## 4 总结与展望

免疫检查点抑制剂在SCLC治疗中的初步研究结果令人鼓舞，考虑到CTLA-4阻断剂和一线化疗联合策略以及PD-1和CTLA-4联合策略的持久反应，有必要进一步探索双靶向免疫治疗联合一线治疗SCLC的方法。当然，免疫检查点抑制剂治疗SCLC也存在原发耐药和获得性耐药，同时，免疫抑制剂治疗带来的毒副作用是临床实践不能忽视的严峻问题。所以，如何有效筛选出对免疫抑制剂可能作出良好反应的患者尤为重要。因SCLC的免疫微环境与其他肿瘤不同，除PD-L1和TMB等研究较多的预测标志物外，关于高微卫星不稳定性（high microsatellite instability, MSI-H）、T细胞炎性基因表达谱（gene expression profile, GEP）和错配修复（mismatch repair, MMR）基因等方面的研究也陆续开展。美国默克公司研究人员评估了利用TMB和T细胞炎性GEP联合预测派姆单抗作出临床反应的潜力，结果显示联合二者可以将肿瘤患者分为对派姆单抗不同临床反应的组群，并鉴定与这些组相关的可靶向的生物学特征模式^[39]^。这种方法可能为合理构建和评估基于免疫检查点抑制剂组合治疗方案提供精准医疗框架，实现个体化免疫治疗。
